# White matter functional networks in the developing brain

**DOI:** 10.3389/fnins.2024.1467446

**Published:** 2024-10-23

**Authors:** Yali Huang, Charles M. Glasier, Xiaoxu Na, Xiawei Ou

**Affiliations:** ^1^Department of Radiology, University of Arkansas for Medical Sciences, Little Rock, AR, United States; ^2^Department of Pediatrics, University of Arkansas for Medical Sciences, Little Rock, AR, United States; ^3^Arkansas Children’s Research Institute, Little Rock, AR, United States; ^4^Arkansas Children’s Nutrition Center, Little Rock, AR, United States

**Keywords:** RS-fMRI, white matter, functional connectivity, fALFF, brain development

## Abstract

**Background:**

Functional magnetic resonance imaging (fMRI) is widely used to depict neural activity and understand human brain function. Studies show that functional networks in gray matter undergo complex transformations from neonatal age to childhood, supporting rapid cognitive development. However, white matter functional networks, given the much weaker fMRI signal, have not been characterized until recently, and changes in white matter functional networks in the developing brain remain unclear.

**Purpose:**

Aims to examine and compare white matter functional networks in neonates and 8-year-old children.

**Methods:**

We acquired resting-state fMRI data on 69 full-term healthy neonates and 38 healthy 8-year-old children using a same imaging protocol and studied their brain white matter functional networks using a similar pipeline. First, we utilized the ICA method to extract white matter functional networks. Next, we analyzed the characteristics of the white matter functional networks from both time-domain and frequency-domain perspectives, specifically, intra-network functional connectivity (intra-network FC), inter-network functional connectivity (inter-network FC), and fractional amplitude of low-frequency fluctuation (fALFF). Finally, the differences in the above functional networks’ characteristics between the two groups were evaluated. As a supplemental measure and to confirm with literature findings on gray matter functional network changes in the developing brain, we also studied and reported functional networks in gray matter.

**Results:**

White matter functional networks in the developing brain can be depicted for both the neonates and the 8-year-old children. White matter intra-network FC within the optic radiations, corticospinal tract, and anterior corona radiata was lower in 8-year-old children compared to neonates (*p* < 0.05). Inter-network FC between cerebral peduncle (CP) and anterior corona radiation (ACR) was higher in 8-year-olds (*p* < 0.05). Additionally, 8-year-olds showed a greater distribution of brain activity energy in the high-frequency range of 0.01–0.15 Hz. Significant developmental differences in brain white matter functional networks exist between the two group, characterized by increased inter-network FC, decreased intra-network FC, and higher high-frequency energy distribution. Similar findings were also observed in gray matter functional networks.

**Conclusion:**

White matter functional networks can be reliably measured in the developing brain, and the differences in these networks reflect functional differentiation and integration in brain development.

## 1 Introduction

Human brain undergoes significant structural and functional changes to support the development of behavior and cognition from infancy to childhood. Based on fMRI, previous studies have shown that functional brain networks undergo complex transformation during this time in the gray matter (Edde et al., 2021; Chen et al., 2021; Bruchhage et al., 2020). While blood oxygenation level-dependent (BOLD) signals have been reliably detected in gray matter using fMRI, and recent studies have shown that neural activity can also be detected in white matter as BOLD signals (Ding et al., 2018; Gore et al., 2019; Huang Y. et al., 2020), research on white matter functional networks in the developing brain has not been reported.

In neonates, the focus of brain function study has been primarily on fundamental autonomic processes and sensory processing (Huang Z. et al., 2020; Gao et al., 2017). In contrast, school age children exhibit a repertoire of more sophisticated cognitive functions, encompassing language, memory, attention, and problem-solving abilities. During this developmental window, there is a clear progression in brain size and volume (Bethlehem et al., 2022). Structural differences between brains of neonates and school age children are evident and have been extensively documented in the literature (Gilmore et al., 2018; Alex et al., 2023). Concomitant with these structural changes, brain function also undergoes substantial transformations. For example, Gao et al. utilized heat maps to quantify the developmental changes in functional connectivity across the first six years of life (Chen et al., 2021). Previous research has also shown that functional connectome can be employed to characterize individual traits (Wang et al., 2021) and to predict age (Kardan et al., 2022). However, these studies have all focused on gray matter regions and have not investigated white matter functional networks.

The goal of this study is to demonstrate that white matter functional networks can be depicted in the developing brain and to investigate the differences in white matter functional networks in different age groups. We hypothesize that there are functional network differences in white matter associated with age. To test this hypothesis, we utilized resting-state fMRI (RS-fMRI) data from two cohorts of children: full term healthy neonates and healthy 8-year-old children and studied and compared their brain white matter functional networks based on three perspectives: functional connectivity within networks (intra-network FC), functional connectivity between networks (inter-network FC), and fractional amplitude of low frequency fluctuations (fALFF) of functional networks. RS-fMRI data for the two groups were acquired using a same imaging protocol on the same 3T MRI scanner and decomposed into independent components using spatial independent component analysis (ICA) (Calhoun et al., 2001) using a similar processing pipeline.

## 2 Materials and methods

### 2.1 MRI data acquisition and pre-processing

We acquired and analyzed two sets of research MRI data, one from full term healthy neonates and one from healthy 8-year-old children. The demographic information of the research subjects is summarized in Table 1, and there was no difference in sex between the two groups (*p* > 0.05). Informed consent was obtained from all subjects following Institutional Review Board approved study protocols at Arkansas Children’s Hospital. Anonymized data were used for analysis.

**Table 1:** Demographic information for the research subjects included in this study.

		Mean	Std.	Range
Neonates	Gestational age (days)	275	6	258–290
	Postnatal age at MRI (days)	20	8	10–54
	Postmenstrual age at MRI (days)	295	11	274–339
	Gender	N/A	N/A	40 M/29 F
8-year-olds	Age at MRI (years)	8.1	0.2	7.9–8.7
	Gender	N/A	N/A	20 M/18 F

The neonates and 8-year-old children’s MRI data were both acquired using a 3.0 T Prisma (Siemens healthcare) scanner. The neonatal scans were performed when they were at natural sleep with no sedation. The 8-year-old children were instructed to close their eyes during the RS-fMRI data acquisition. The MRI protocol included a MPRAGE 3D T1-weighted scan with TR 2400ms, TE 2.24ms, 1 average, voxel size 0.8*0.8*0.8mm^3^, 8° flip angle, turbo factor 256, and sagittal slices covering the entire brain, and a RS-fMRI pulse sequence with multi-band EPI with TR 800ms, TE 37ms, phase-encoding AP direction then repeated in PA direction, multi-band factor 8, matrix size 104*104, voxel size 2*2*2 mm^3^, and 72 slices. In addition, a short field map sequence using the same geometry was performed for both AP/PA phase encoding, to correct for field inhomogeneity associated distortion in imaging post-processing.

The 8-year-old children’s brain structural and functional images were preprocessed by the fMRIPrep toolbox (Esteban et al., 2019), which includes tools from FSL (Jenkinson et al., 2012), AFNI (Cox, 1996), and ANTs (Avants et al., 2009). The pipeline in our study includes (1) slice timing correction; (2) correction for susceptibility distortions induced by magnetic field inhomogeneity and multi-band EPI; (3) realignment of all volumes to a selected reference volume; (4) co-registration of the functional data to the structural image; (5) normalization to the MNI standard space, data resampling to 2*2*2 mm^3^ isotropic voxels; (6) functional images’ segmentation into gray matter and white matter; and (7) smoothing the segmented white matter with a 4 mm FWHM Gaussian kernel.

Since the fMRIPrep toolbox by default lacks an age-appropriate template for the neonatal brain, we applied in-house code base on FSL, AFNI and ANTs, same as those used in fMRIPrep, to perform data preprocessing on neonates (Jenkinson et al., 2012; Cox, 1996; Avants et al., 2009). It should be noted that all steps were the same as those used in fMRIPrep and the only difference between processing of the neonatal and 8-year-old data was that the neonatal data were registered to the UNC neonatal template (Shi et al., 2011) whereas data for 8-year-old children were registered to the MNI space. And the white and gray matter masks from UNC were used to segment the white and gray matter data for the neonates.

Head motion of all subjects was checked, and only subjects with RS-fMRI data with minimal to slight head motion (<3.0 mm in translation and < 3° in rotation for all imaging volumes for 8-year-old children; <2.0 mm in translation and < 2° in rotation for all imaging volumes for neonates) were kept in the study. After the head motion evaluation, 3 subjects from the 8-year-old group were excluded and 38 subjects were used for further analysis; and 3 subjects from the neonatal group were excluded and 69 subjects were used for further analysis.

### 2.2 Group ICA parcellation

We first decomposed the two preprocessed BOLD data separately using standard group-level ICA to identify statistically ICs, as implemented in the GIFT toolbox.^1^ The data were decomposed using the Infomax ICA algorithm, and the ICASSO algorithm was repeated 20 times to increase the stability of ICs. Subject-specific spatial maps and time courses were estimated using the GICA back-reconstruction method, similar to that performed in previous research on whole-brain connectivity in the resting state (Allen et al., 2014). FMRI data of white matter were decomposed into 16 and 20 independent components for the neonates and 8-year-old children, respectively. Eight year old children have a more mature brain network development compared to neonates, and their BOLD signal were therefore decomposed to a larger number of independent components, consistent with previous studies (Gao et al., 2015b). A similar processing scheme was also applied to the gray matter fMRI data for references.

### 2.3 Identification of functional networks

The group-average spatial maps obtained from the GIFT output were visually inspected, with each component manually labeled as either a functional network or noise. Following the method of functional network identification in gray matter ICA decomposition by Du et al. (2023), we organized the spatial maps from the white matter ICA decomposition into specific functional networks. To determine whether a component constituted a meaningful functional network and should be included in the analysis, we removed components located on the scalp or blood vessels, compared each component to previous research findings (Gao et al., 2017; Gilmore et al., 2018; Allen et al., 2014; Allen et al., 2011; Seeley, 2019; Du et al., 2020), and identified white matter functional networks according to their anatomical roles and the JHU atlas (Du et al., 2023; Allen et al., 2011).

**Figure 1:**
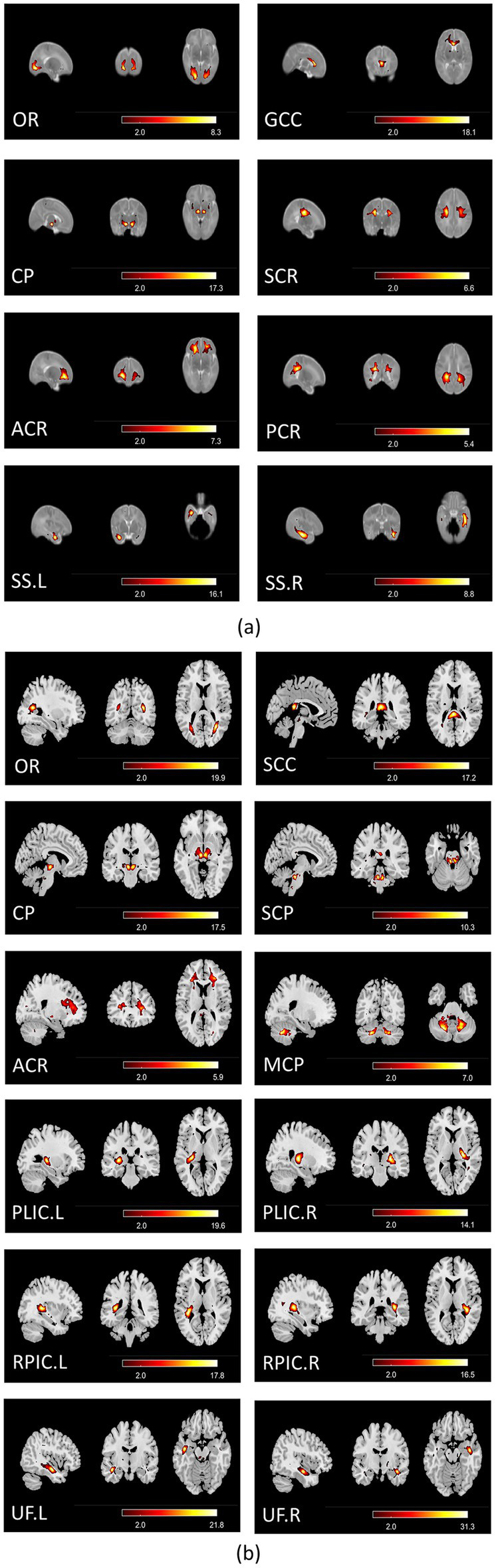
(a) Group-average spatial maps for white matter functional networks in neonates; (b) group-average spatial maps for white matter functional networks in 8-year-old children. All spatial maps were thresh-hold at Z > 2 (*p* < 0.05), and the color bar denotes Z-scores. OR, Optic Radiation; CP, Cerebral Peduncle; ACR, Anterior Corona Radiata; SCR, Superior Corona Radiata; PCR, Posterior Corona Radiata; GCC, Genu of Corpus Callosum; SS, Sagittal Stratum; SCC, Splenium of Corpus Callosum; MCP, Middle Cerebellar Peduncle; SCP, Superior Cerebellar Peduncle; PLIC, Posterior Limb of Internal Capsule; RPIC, Retrolenticular Part of Internal Capsule; UF, Uncinate Fasciculus.

For the comparison of functional networks between the two age groups, since the functional brain networks of neonates are still developing and not fully mature (Huang Z. et al., 2020; Gao et al., 2015b; Gao et al., 2015a), it may be challenging to establish a one-to-one correspondence between the independent components obtained from neonates and those obtained for 8-year-old children. We focused on optic radiation (OR), cerebral peduncle (CP) and anterior corona radiation (ACR) functional networks in both datasets, which are consistently delineated in both groups (Figure 1) and are essential for the visual, motor, and cognitive functions. For gray matter, we identified the primary functional networks including the visual network (VN), the sensorimotor network (SMN), and the auditory network (AN), as well as high-order networks including the default mode network (DMN), the salience network (SAN), and the frontoparietal networks (FPN) in both datasets (Supplementary Figure S1).

### 2.4 Computation of intra-network FC and inter- network FC

To remove the influence of irrelevant signals, we conducted the following post-processing on all time courses (TC): transforming TC to a Fisher’s Z-score, regressing six head motion parameters, and removing linear trends. This analytical framework is systematically applied to both white matter and gray matter fMRI data sets respectively, ensuring a comprehensive understanding of FC across different brain tissue types.

Consequently, we analyzed the differences in intra-network and inter-network FCs among the 3 white matter functional networks and 6 gray matter functional networks between the two age groups. As an additional measure, we also examined the differences in both intra-network FC and inter-network FC for all meaningful functional networks (as presented in Figure 1) combined, regardless of whether they were consistently detected in both age groups.

For each functional network, we assessed intra-network FC by computing the mean Pearson correlation coefficient across the voxel TC within the network, excluding diagonal elements. For each participant, we also calculated the average intra-network FC for all functional networks to derive the intra-network FC. The main objective of intra-network FC is to assess the synchronous activity within the functional network. Positive correlations reflect the temporal consistency of activities among different voxels within the network, which is crucial for measuring the network’s homogeneity. Negative correlations, on the other hand, might indicate functional differentiation within the network, which is not aligned with our purpose of assessing homogeneity. In brain functional studies, positive correlations are often viewed as indicators of functional connectivity, representing regions that are either simultaneously activated or inhibited. Negative correlations are less commonly used to denote such synchronous activity, especially when evaluating connectivity within a single network. Therefore, for intra-network FC, we only considered positive correlations within the network.

For each participant, we computed the inter-network FC by evaluating the mean Pearson correlation coefficient matrix among all pairs of the identified functional networks, again omitting diagonal elements. This process provides us with distinct inter-network FC values for each individual. For inter-network FC, we include both positive and negative correlations between networks. The goal of inter-network FC is to assess the interactions between different functional networks. Positive correlations indicate cooperative activity between networks, while negative correlations represent a complementary or inhibitory relationship. Both types of correlations are biologically significant in the context of inter-network interactions. Considering the complex interaction patterns between different networks, including both positive and negative correlations ensures a more comprehensive evaluation of inter-network functional connectivity. Omitting negative correlations would overlook crucial information about the nature of these interactions. Averaging both positive and negative correlations to evaluate overall inter-network functional connectivity is commonly used in clinical and cognitive neuroscience research, especially in studies that require a comprehensive assessment of network connectivity strength or changes in connectivity patterns (Berman et al., 2016; Sahoo et al., 2015; Song et al., 2015). Therefore, we considered both positive and negative inter-network FC.

### 2.5 Investigation of frequency spectrum in functional networks

The amplitude of low-frequency fluctuation (ALFF) of the resting-state fMRI signal has been suggested to reflect the intensity of regional spontaneous brain activity (Zang et al., 2007). Due to its sensitivity to noise, the fALFF was subsequently proposed to study brain function (Zou et al., 2008). Here we calculated the ratio of power spectrum at 11 frequency bands to that of the frequency range (0.01 ~ 0.15 Hz), which denote the power of BOLD signal fluctuation. These 11 frequency bands starting from 0.01 Hz to 0.15 Hz, each frequency band is 0.04 Hz in length, with a step size of 0.01 Hz. For each independent component of each subject, TC was extracted to compute fALFF at the 11 frequency bands. Similar to the process used in calculating FC, we also applied the following post-processing to TC: Fisher-Z score transformation, head-motion regression, and detrending before computing fALFF.

## 3 Results

### 3.1 Identification of white matter functional networks using ICA

The identified white matter functional networks for neonates are as follows: OR (Optic Radiation), GCC (Genu of Corpus Callosum), CP (Cerebral Peduncle), SCR (Superior Corona Radiata), ACR (Anterior Corona Radiata), PCR (Posterior Corona Radiata), and SS (Sagittal Stratum), as shown in Figure 1a. The identified white matter functional networks for 8-year-old children are as follows: OR (Optic Radiation), SCC (Splenium of Corpus Callosum), CP (Cerebral Peduncle), SCP (Superior Cerebellar Peduncle), ACR (Anterior Corona Radiata), MCP (Middle Cerebellar Peduncle), PLIC (Posterior Limb of Internal Capsule), RPIC (Retrolenticular Part of Internal Capsule), and UF (Uncinate Fasciculus), as shown in Figure 1b. Possibly due to the immaturity of functional networks in neonates and the much lower BOLD signal in white matter, only three networks: OR, CP and ACR were consistently identified (the first three rows of the left column in Figures 1a,b) and were used for group comparison.

### 3.2 Intra-network FC analysis in white matter between two groups

**Figure 2:**
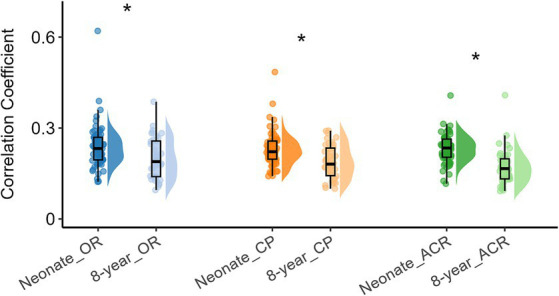
Difference in intra-network FC within white matter functional networks (including OR, CP and ACR) in neonates and 8-year-old children. Asterisks above the box plots indicate significant differences (*p* < 0.05, FDR corrected) between neonates and 8-year-old children (two-sample T-test).

To investigate how functional networks evolve from neonates to 8-year-old children, we compared the intra-network FC of the above three networks, with the corresponding results presented in Figure 2, which shows that the intra-network FC within OR, CP, and ACR were lower (all *p* values <0.05, FDR corrected) when compare 8-year-old children to neonates.

**Figure 3:**
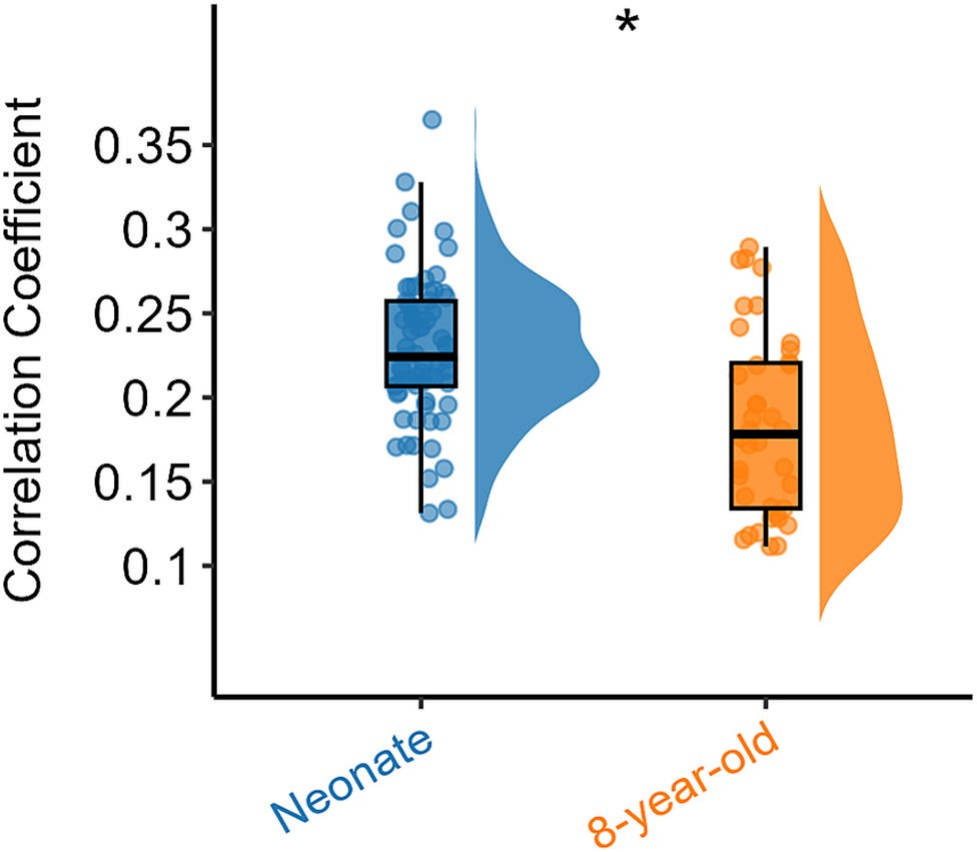
Overall intra-network white matter FC differences between the two age groups. Asterisks above the box plots indicate significant differences (*p* < 0.05) between neonates and 8-year-old children (two-sample T-test).

To explore the differences in intra-network FC of all white brain networks between neonates and 8-year-old children from a holistic perspective, we further examined the overall intra-network FC within white matter for all functional networks derived from the ICA algorithm (Figure 1) for the two age groups, and the results are shown in Figure 3. It can be also seen that the intra-network FC was lower in 8-year-old children compared to that in neonates. These results suggest that with an increase in age, the intra-network FC within the same functional networks decreases.

### 3.3 Inter-network FC analysis in white matter between two groups

**Figure 4:**
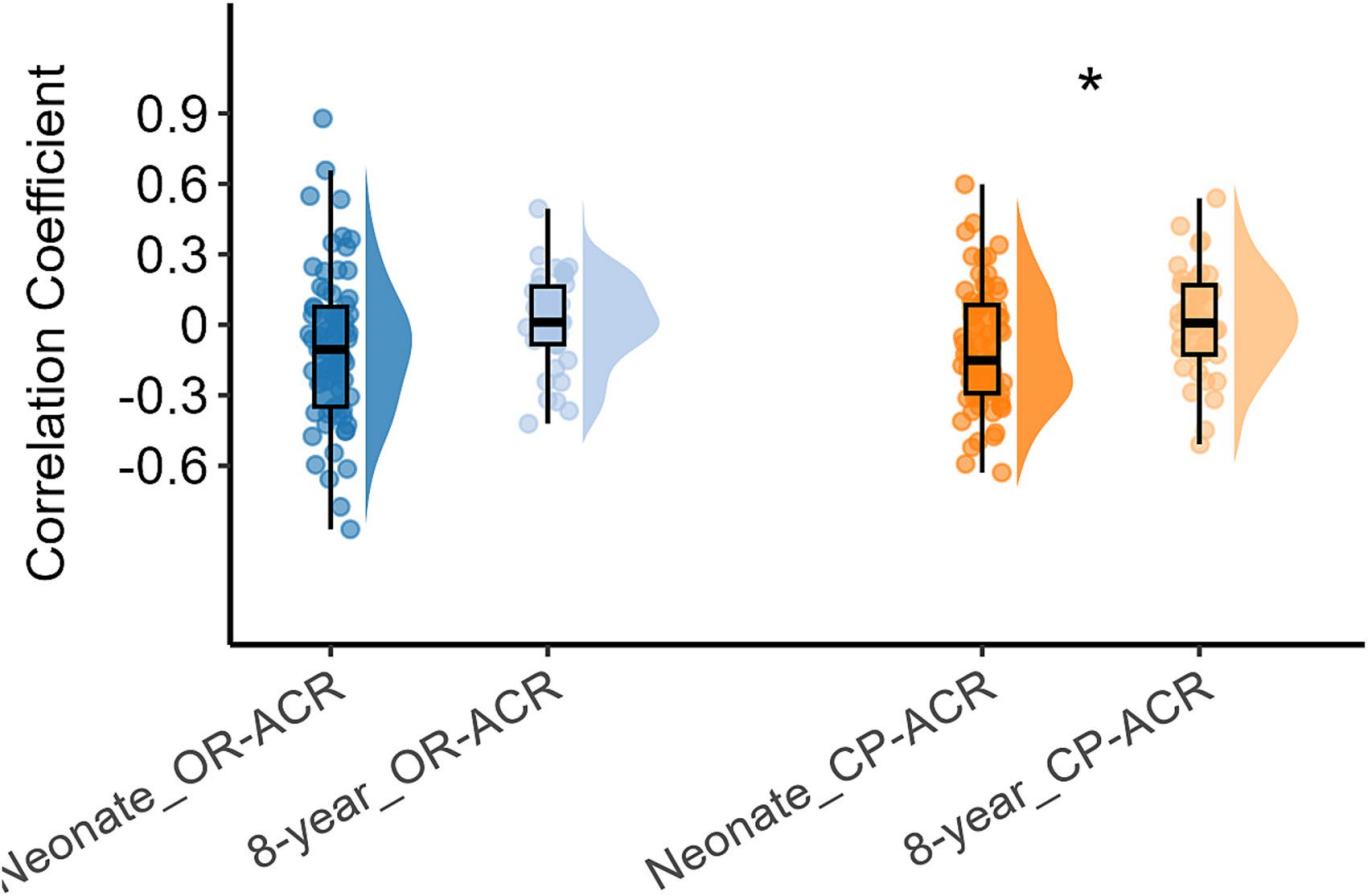
Differences in inter-network FC between white matter functional networks (including OR, CP, and ACR) in neonates and 8-year-old children. Asterisks above the box plots indicate significant differences (*p* < 0.05) between neonates and 8-year-old children (two-sample T-test).

Next, we compared the inter-network FC of the three networks between neonates and 8-year-olds, with the corresponding results presented in Figure 4, which indicated that the inter-network FC between CP and ACR were higher when compare 8-year-old children to neonates (*p* < 0.05), while the inter-network FC between OR and ACR were similar between the two age groups (*p* = 0.08). Here we mainly measure the inter-networks between basic and advanced networks, so we did not present the inter-networks FC between OR and CP (which are both basic brain functional networks).

**Figure 5:**
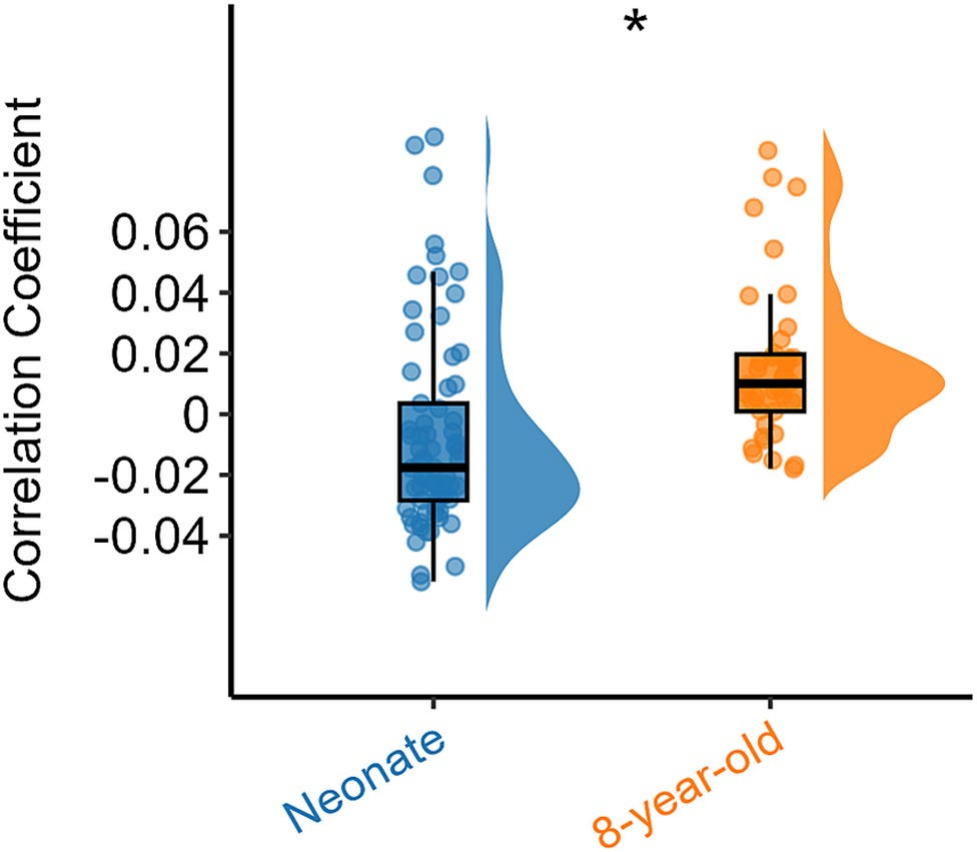
Overall inter-network white matter FC differences between the two age groups. Asterisks above the box plots indicate significant differences (*p* < 0.05) between neonates and 8-year-old children (two-sample T-test).

Similarly, we also examined the overall inter-network FC within white matter for all functional networks using the ICA algorithm (Figure 1) and compared them between the two age groups, and the results are shown in Figure 5. It can be also seen that the inter-network FC was higher in 8-year-old children compared to those in neonates. These results suggest that with an increase in age, the inter-network FC between the different functional networks increases.

### 3.4 Functional networks fALFF analysis in white matter between two groups

**Figure 6:**
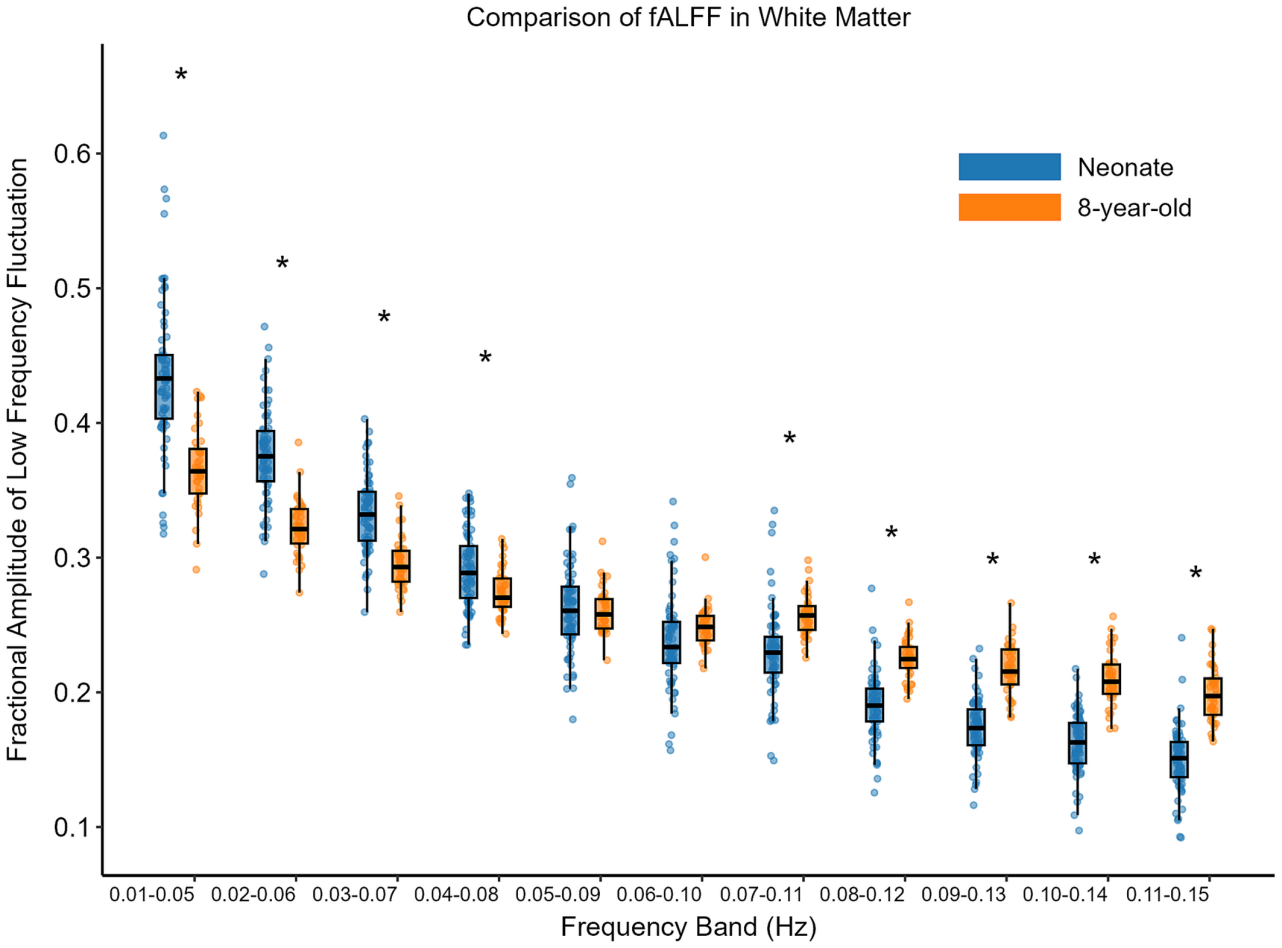
Comparison of fALFF between neonates and 8-year-old in white matter. The horizontal axis shows 11 different frequency bands; the vertical axis represents fALFF. The box on the left above each frequency band represents neonates, while the one on the right represents 8-year-old children. Asterisks above the box plots indicate significant differences (*p* < 0.05, FDR corrected) between neonates and 8-year-old children (two-sample T-test).

Firstly, we calculated the fALFF of each component for each participant (excluding noise components). Then, we averaged the fALFF of all components for each participant to obtain the fALFF of that participant in a specific frequency band. Finally, we compared the fALFF across different frequency bands between neonates and 8-year-old children. Multiple comparison corrections (FDR) were applied to account for the 11 frequency bands. The comparison of fALFF in white matter functional networks is presented in Figure 6. Specifically, in the low-frequency range (0.01–0.05 Hz, 0.02–0.06 Hz, 0.03–0.07 Hz, 0.04–0.08 Hz), the fALFF for the 8-year-old children were lower than those for the neonates (*p* < 0.05, FDR corrected); and in higher frequency bands (0.07–0.11 Hz, 0.08–0.12 Hz, 0.09–0.13 Hz, 0.10–0.14 Hz, 0.11–0.15 Hz), the 8-year-old children had higher fALFF than the neonates (*p* < 0.05, FDR corrected).

We also found similar results in the gray matter functional network: the intra-network FC of 8-year-old children is lower than that of newborns, while the inter-network FC is higher than that of newborns. Additionally, with increasing age, the distribution of fALFF shifts toward higher frequencies. Detailed results can be found in the Supplementary material.

## 4 Discussion

We conducted an investigation of brain functional networks in both the temporal domain and the frequency domain to examine the differences in white matter functional networks between neonates and 8-year-old children. Traditional functional network research has predominantly concentrated on gray matter, since white matter BOLD signal is much weaker, and it has not been studied extensively until recently (Gore et al., 2019; Li et al., 2019; Wang et al., 2022; Huang et al., 2023). We expanded current literature findings to explore the white matter functional networks in the developing brain based on RS-fMRI data.

In the FC analysis, we approached the investigation from two perspectives. First, we analyzed the differences in both intra- and inter-network FC within specific functional networks that were consistently presented in both neonates and 8-year-old children. Then, we examined the intra- and inter-network FC differences across all meaningful functional networks identified by ICA, regardless of whether they were consistently present in the two age groups. For both approaches, our findings show lower white matter intra-network FC and higher white matter inter-network FC in 8-year-old children compared to neonates. These findings are consistent with gray matter functional network findings presented in the Supplementary material, which also showed lower intra-network FC and higher inter-network FC in 8-year-old children compared to neonates for primary functional networks (including VN, SMN, and AN) and higher-order functional networks (including DMN, SAN, and FPN) and for all functional networks. Combined, our results reflect a differentiation of brain function within the same networks, and an integration of different functional networks with age. This developmental pattern is consistent with reports of gray matter functional connectivity in children by other studies (Edde et al., 2021; Gao et al., 2015b; Betzel et al., 2014; Chan et al., 2014). Specifically, previous studies demonstrate that there is a strong and widespread organization of networks, starting with segregation processes followed by a continuous increase in integration during the first year of the life, followed by a refinement of existing functional networks, which are characterized by an increase in integrative processes until about 40 years of age (Edde et al., 2021). Gao et al. (2015b) reported that increased FC is predominantly observed among higher-order cognitive function networks. Other studies indicated that the adult brain also displays reduced within-network and increased between-network FC across the adult lifespan (Du et al., 2023; Betzel et al., 2014; Chan et al., 2014; Deery et al., 2023). Adaptive changes in FC between different networks with age may be one of the contributing factors to the evolvement of higher-order cognitive functions during human life span.

Previous studies also showed that BOLD effects in white matter are robustly detectable both in response to stimuli and in a resting state, which have been largely ignored in previous fMRI literature (Huang Z. et al., 2020). Recently, researchers have explored biomarkers for depression and children with attention-deficit disorder based on white matter BOLD signals (Li et al., 2020; Bu et al., 2020). However, there are few reports on white matter BOLD signals associated with child brain development. Our research results indicate significant differences in the white matter functional networks between 8-year-old children and newborns. These differences in functional network connectivity signify adaptive adjustments in brain function from infancy to childhood, e.g., neonates predominantly manifest basic physiological rhythms in their brainwave activity, whereas 8-year-old children already exhibit more intricate and advanced cognitive functions in their brainwave patterns. This cognitive evolution is underpinned by the increasing complexity and refinement of neural connections within the brain, including both white matter and gray matter. This enhanced connectivity allows for improved coordination and efficient exchange of information between different regions of the brain (Edde et al., 2021; Bruchhage et al., 2020; Bethlehem et al., 2022). It should be noted that different FC results can be obtained when using different band-pass filters. In our study, the FC values are based on the same band-pass filtering within the 0.01–0.15 Hz range.

The fALFF is an important indicator of brain functional activity. Our study reveals that in the lower frequency range of 0.01–0.15 Hz, 8-year-old children exhibited lower fALFF compared to neonates. In contrast, at higher frequency ranges, 8-year-old children showed higher fALFF than neonates. This was true for both white matter and gray matter. Our results of gray matter align with previous research findings that there is a clear rightward shift in the BOLD signal frequency during the first year of life (Alcauter et al., 2015). Our results also suggest that this rightward shift in the BOLD signal spectrum is more prominent in white matter functional networks. This result is consistent with the findings of a previous study that simple functional connectivity is predominantly occupied by low-frequency BOLD oscillations, while complex functional connectivity networks are mainly occupied by high-frequency BOLD oscillations (Baria et al., 2011). Therefore, the development of higher-order cognitive functions during brain maturation may result in the differences in fALFF.

Despite its novelty in evaluation of brain functional networks and activity at rest in white matter, our study has certain limitations. First, the sample size is relatively small. Second, our study is cross-sectional but not longitudinal and there is a lack of data encompassing the developmental stages in between neonates and 8-year-olds. Existing and future data from large, multi-center, and longitudinal neuroimaging studies may be able to address this limitation, confirm our findings, and characterize brain resting-state functional network changes throughout all brain developmental stages. Third, the different states of newborns and 8-year-old children during MRI data collection may have influenced our results. Several studies have investigated the differences in FC between sleep and awake states. Research indicates that while there are notable differences in brain activity patterns between these states, the core networks and their functional connectivity tend to be preserved. For example, studies have shown that key resting-state networks, such as the default mode network (DMN), remain identifiable both during sleep and wakefulness, albeit with variations in their connectivity strengths and dynamics (Horovitz, et al., 2009; Larson-Prior et al., 2011). Horovitz et al. (2009) found that although the DMN exhibits reduced connectivity during deep sleep stages, its presence is still discernible. Similarly, Larson-Prior et al. (2011) demonstrated that the fundamental structure of resting-state networks remains intact during different sleep stages, even though their connectivity patterns are modulated by the level of consciousness. It is speculated that the differences associated with sleep and awake states, while present, are less significant compared to the substantial developmental changes in brain connectivity from neonates to 8-year-olds. Age-related differences in brain development involve extensive maturation and reorganization of neural networks, which likely have a more profound impact on FC than the transient state differences between sleep and wakefulness. Therefore, while we acknowledge the potential influence of sleep versus awake states on our results, we believe that the age-related developmental changes we observe are the predominant factor. Nonetheless, future studies with more standardized data collection procedures across different age groups and states of consciousness will be beneficial to further validate our findings.

## 5 Conclusion

Our study indicates that white matter functional networks can be depicted in the developing brain using resting-state fMRI, and there are significant differences in white matter brain functional networks between neonates and 8-year-old children from three measures (intra-networks FC, inter-networks FC and fALFF). This phenomenon, indicative of the brain’s adaptive developmental processes, is evident in white matter and is consistent with the patterns observed in gray matter by us and by others as well. The analysis of FC and fALFF in white matter brain’s functional networks between neonates and 8-year-old children can contribute to better understanding of normal child brain development.

## Data Availability

The raw data supporting the conclusions of this article will be made available by the authors, without undue reservation.

## Footnotes

^1^ https://trendscenter.org/software/gift/
