# Supplementary material for: White matter functional networks in the developing brain
Source: Front Neurosci. 2024 Oct 23;18:1467446. doi: 10.3389/fnins.2024.1467446 (PMC11538026; doi:10.3389/fnins.2024.1467446)
Supplement: Supplementary file 1 [file Data_Sheet_1.docx]

Supplementary Material

# Results on gray matter

## Identification of gray matter functional networks consistently present in both age groups using ICA

Six functional networks were found to be consistently present in both the neonates and 8-year-olds using ICA algorithms, which were the primary functional networks including the visual network (VN), the sensorimotor network (SMN), and the auditory network (AN), as well as high-order networks including the default mode network (DMN), the salience network (SAN), and the frontoparietal networks (FPN) in both datasets (Fig. S1).

## Intra-network FC analysis in gray matter.

We compared the intra-network FC of the six functional networks in the gray matter between the two groups of subjects, as shown in Fig. S2, which shows that the intra-network FC of both primary and higher-order functional networks were lower (all *p* values <0.05, FDR correction) when compare 8-year-old children to neonates.

To investigate the differences in intra-network FC of all brain networks between neonates and 8-year-old children from a holistic perspective, we further examined the overall intra-network FC within gray matter for all functional networks derived from the ICA algorithm (regardless of whether the networks can be identified consistently in both neonate and 8-year-old data) for the two age groups (Fig. S3). The overall intra-network FC was lower in 8-year-old children compared to that in neonates. These results suggest that with an increase in age, the intra-network FC within the same functional networks decreases.

## Inter-network FC analysis in gray matter.

Similarly, we performed a comparable analysis on the inter-network FC of primary and higher-order functional networks in gray matter between the two groups of subjects, as shown in Fig. S4, which shows that the inter-network FC between the primary network (VN, SMN, or AN) and the high-order network (DMN, SAN, or FPN) were higher (*p* values <0.05) when compare 8-year-old children to neonates.

Similarly, we also examined the overall inter-network FC within white matter for all functional networks derived from the ICA algorithm (regardless of whether the networks can be identified consistently in both neonate and 8-year-old data), and compared them between the two age groups (Fig. S5), and it can be seen that the overall inter-network FC was higher in 8-year-old children compared to those in neonates. These results suggest that with an increase in age, the inter-network FC between primary and high-order networks increases.

## fALFF in gray matter functional networks.

For both neonates and 8-year-old children, we calculated the fALFF of each independent component for each individual and compared the fALFF between the two groups (exclude noise component). The comparison of fALFF in the gray matter is presented in Fig. S6. In the lower frequency range (0.01-0.05Hz, 0.02-0.06Hz, 0.03-0.07Hz, 0.04-0.08Hz,), the fALFF for the 8-year-old children were lower than those for the neonates (*p* < 0.05). In the higher frequency bands (0.08-0.12Hz, 0.09-0.13Hz, 0.10-0.14Hz, 0.11-0.15Hz), the 8-year-old children had higher fALFF than the neonates (*p* < 0.05). After FDR correction, the frequency bands with *p* < 0.05 are: 0.03-0.07Hz, 0.08-0.12Hz, 0.09-0.13Hz, 0.10-0.14Hz, and 0.11-0.15Hz.

# Figures

Fig. S1. The left column: group-average spatial maps for gray matter functional networks in neonates. The right column: group-average spatial maps for gray matter functional networks in 8-year-old children. VN: visual network; SMN: sensorimotor network; AN: auditory network; DMN: default mode network; SAN: salience network; FPN: frontoparietal network. All spatial maps were thresholded at Z > 2 (*p* < 0.05), and the color bar denotes Z-scores.

Fig. S2. Differences in intra-network FC within the primary networks (including VN, SMN, and AN) and the high-order networks (including DMN, SAN, and FPN) in gray matter between neonates and 8-year-old children. Asterisks above the box plots indicate significant differences (*p* < 0.05, FDR corrected) between neonates and 8-year-old children (two-sample T-test).

Fig. S3. Overall intra-network gray matter FC differences between the two age groups. Asterisks above the box plots indicate significant differences (*p* < 0.05) between neonates and 8-year-old children (two-sample T-test).

Fig. S4. Differences in inter-network FC between the primary networks (including VN, SMN, and AN) and the high-order networks (including DMN, SAN, and FPN) in gray matter between neonates and 8-year-old children. Asterisks above the box plots indicate significant differences (*p* < 0.05) between neonates and 8-year-old children (two-sample T-test).

Fig. S5. Overall inter-network gray matter FC differences between the two age groups. Asterisks above the box plots indicate significant differences (*p* < 0.05) between neonates and 8-year-old children (two-sample T-test).

Fig. S6 Comparison of fALFF between neonates and 8-year-old in gray matter. The horizontal axis shows 11 different frequency bands; the vertical axis represents fALFF. The box on the left above each frequency band represents neonates, while the one on the right represents 8-year-old children. Asterisks above the box plots indicate significant differences (*p* < 0.05) between neonates and 8-year-old children (two-sample T-test). After FDR correction, the frequency bands with *p* < 0.05 are: 0.03-0.07Hz, 0.08-0.12Hz, 0.09-0.13Hz, 0.10-0.14Hz, and 0.11-0.15Hz.


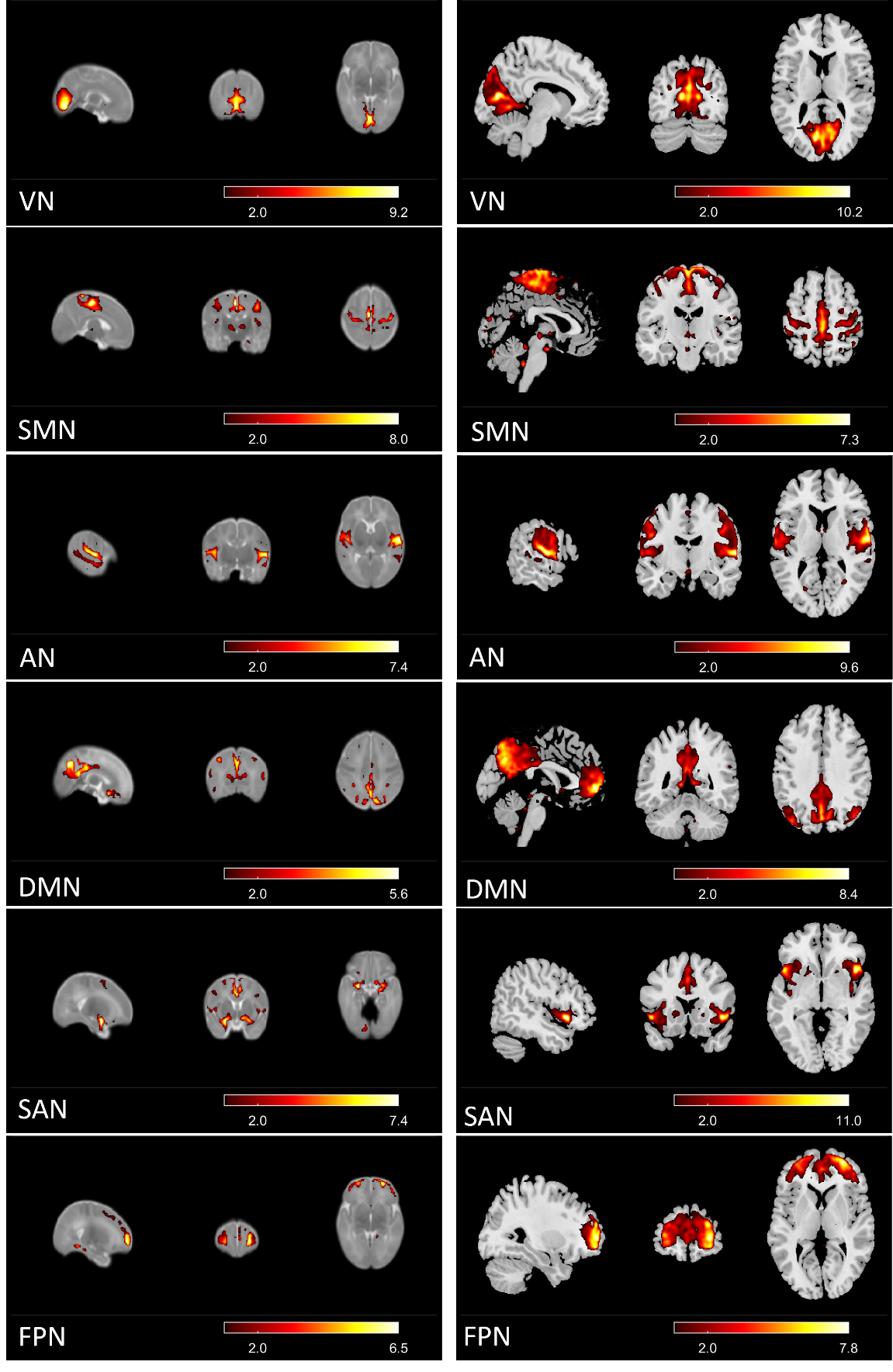


Fig. S1. The left column: group-average spatial maps for gray matter functional networks in neonates. The right column: group-average spatial maps for gray matter functional networks in 8-year-old children. VN: visual network; SMN: sensorimotor network; AN: auditory network; DMN: default mode network; SAN: salience network; FPN: frontoparietal network. All spatial maps were thresholded at Z > 2 (*p* < 0.05), and the color bar denotes Z-scores.


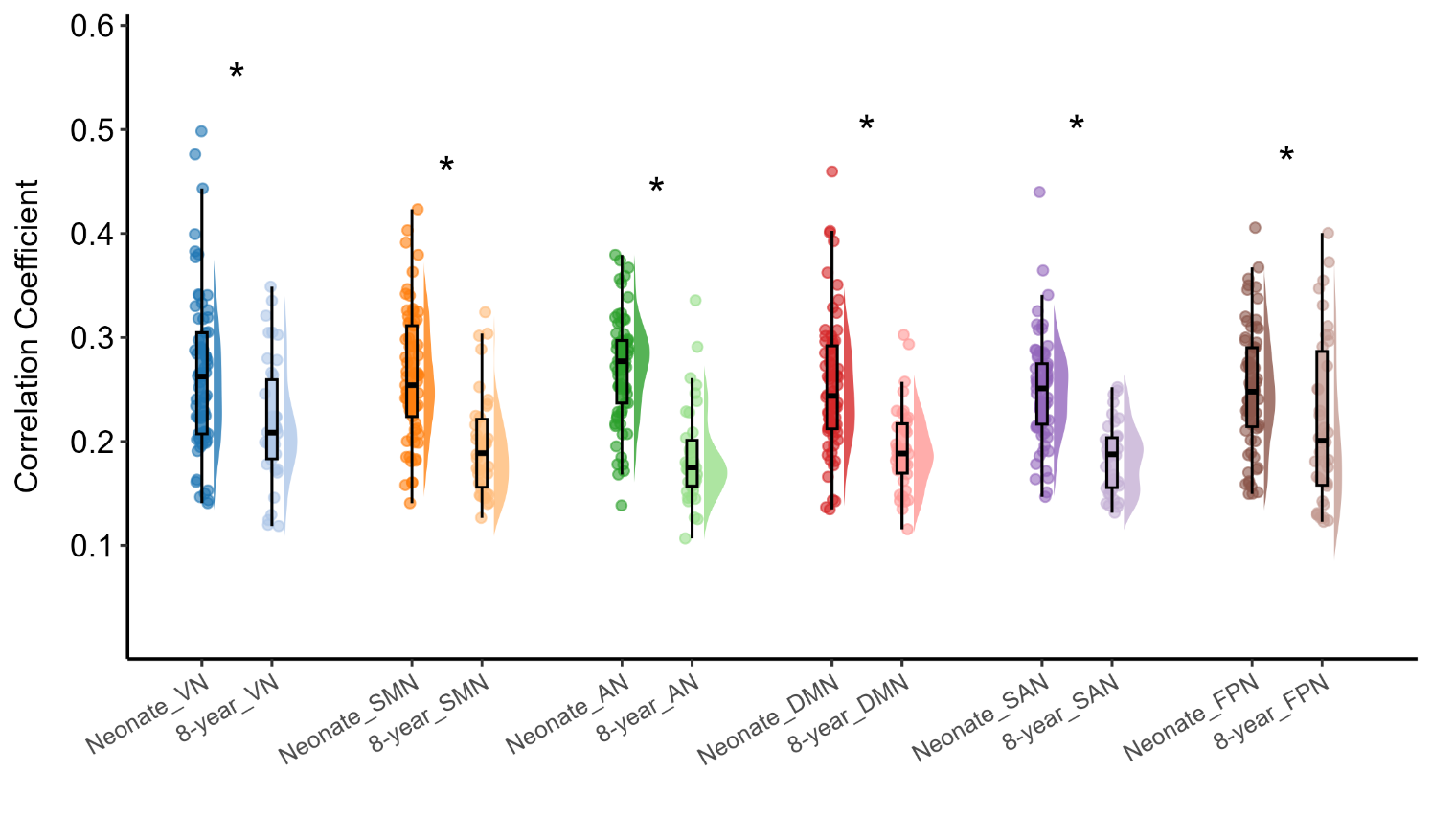


Fig. S2. Differences in intra-network FC within the primary networks (including VN, SMN, and AN) and the high-order networks (including DMN, SAN, and FPN) in gray matter between neonates and 8-year old children. Asterisks above the box plots indicate significant differences (*p* < 0.05, FDR corrected) between neonates and 8-year-old children (two-sample T-test).


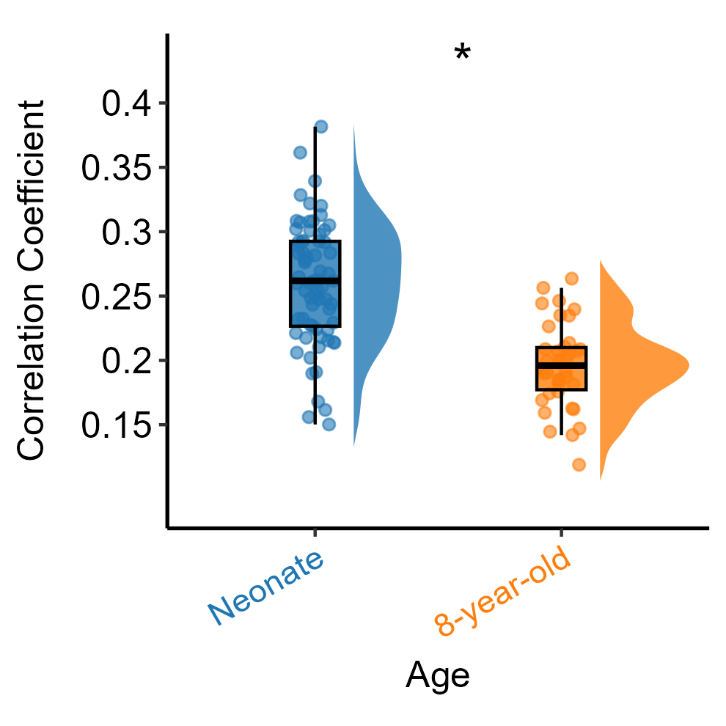


Fig. S3. Overall intra-network gray matter FC differences between the two age groups. Asterisks above the box plots indicate significant differences (*p* < 0.05) between neonates and 8-year-old children (two-sample T-test).


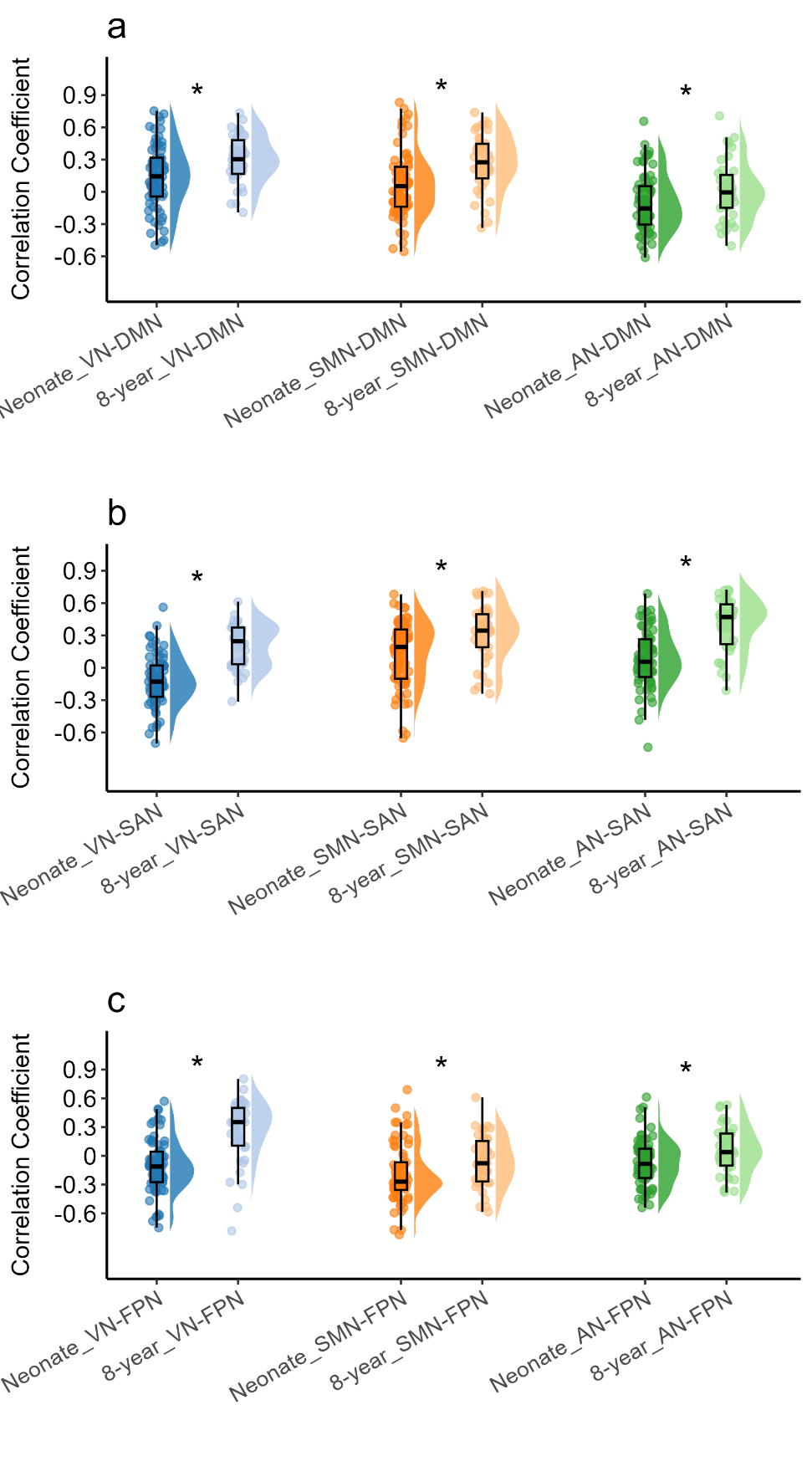


Fig. S4. Differences in inter-network FC between the primary networks (including VN, SMN, and AN) and the high-order networks (including DMN, SAN, and FPN) in gray matter between neonates and 8-year old children. Asterisks above the box plots indicate significant differences (*p* < 0.05) between neonates and 8-year-old children (two-sample T-test).


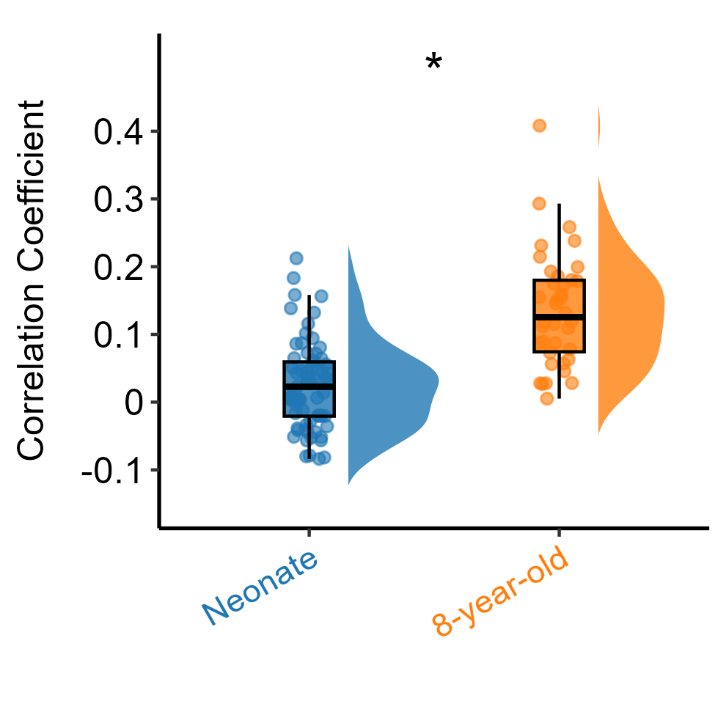


Fig. S5. Overall inter-network gray matter FC differences between the two age groups. Asterisks above the box plots indicate significant differences (*p* < 0.05) between neonates and 8-year-old children (two-sample T-test).


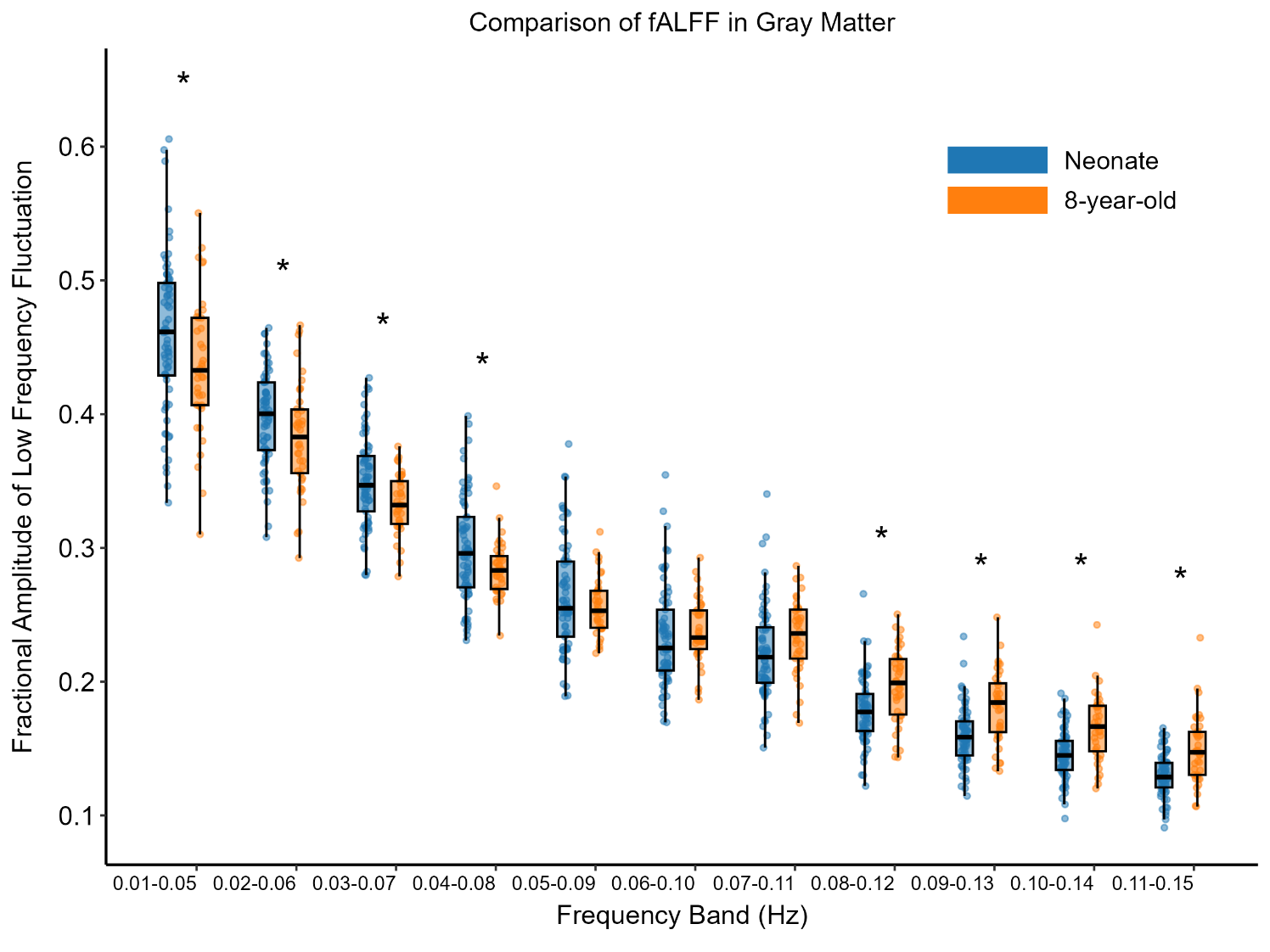


Fig. S6 Comparison of fALFF between neonates and 8-year-old in gray matter. The horizontal axis shows 11 different frequency bands; the vertical axis represents fALFF. The box on the left above each frequency band represents neonates, while the one on the right represents 8-year-old children. Asterisks above the box plots indicate significant differences (*p* < 0.05) between neonates and 8-year-old children (two-sample T-test). After FDR correction, the frequency bands with *p* < 0.05 are: 0.03-0.07Hz, 0.08-0.12Hz, 0.09-0.13Hz, 0.10-0.14Hz, and 0.11-0.15Hz.
